# Mutations in the Serine/Threonine Kinase BRAF: Oncogenic Drivers in Solid Tumors

**DOI:** 10.3390/cancers16061215

**Published:** 2024-03-20

**Authors:** Paola Roa, Nicole Virginia Bremer, Valentina Foglizzo, Emiliano Cocco

**Affiliations:** Sylvester Comprehensive Cancer Center, Department of Biochemistry and Molecular Biology, Miller School of Medicine, University of Miami, Miami, FL 33136, USA; pxr421@miami.edu (P.R.); nvb29@miami.edu (N.V.B.); vxf221@miami.edu (V.F.)

**Keywords:** BRAF, solid tumors, NGS, targeted therapy, therapy resistance

## Abstract

**Simple Summary:**

In this literature review, we explore the milestone events from the discovery of *BRAF* mutations to present-day clinical intervention strategies. We delve into the role of the *BRAF* gene in various cancer types such as melanoma, non-small-cell lung cancer, colorectal cancer, and thyroid cancer. Additionally, we reviewed clinical trials that led to the FDA approval of therapeutic regimens as monotherapy or, more recently, as combinatorial approaches to treat cancer types harboring *BRAF* hotspot mutations.

**Abstract:**

Since their discovery in 2002, *BRAF* mutations have been identified as clear drivers of oncogenesis in several cancer types. Currently, their incidence rate is nearly 7% of all solid tumors with BRAF V600E constituting approximately 90% of these diagnoses. In melanoma, thyroid cancer, and histiocytic neoplasms, BRAF hotspot mutations are found at a rate of about 50%, while in lung and colorectal cancers they range from 3% to 10% of reported cases. Though present in other malignancies such as breast and ovarian cancers, they constitute a small portion of diagnoses (<1%). Given their frequency along with advancements in screening technologies, various methods are used for the detection of BRAF-mutant cancers. Among these are targeted next-generation sequencing (NGS) on tumor tissue or circulating tumor DNA (ctDNA) and immunohistochemistry (IHC)-based assays. With advancements in detection technologies, several approaches to the treatment of BRAF-mutant cancers have been taken. In this review, we retrace the milestones that led to the clinical development of targeted therapies currently available for these tumors.

## 1. Introduction

**Figure 1:**
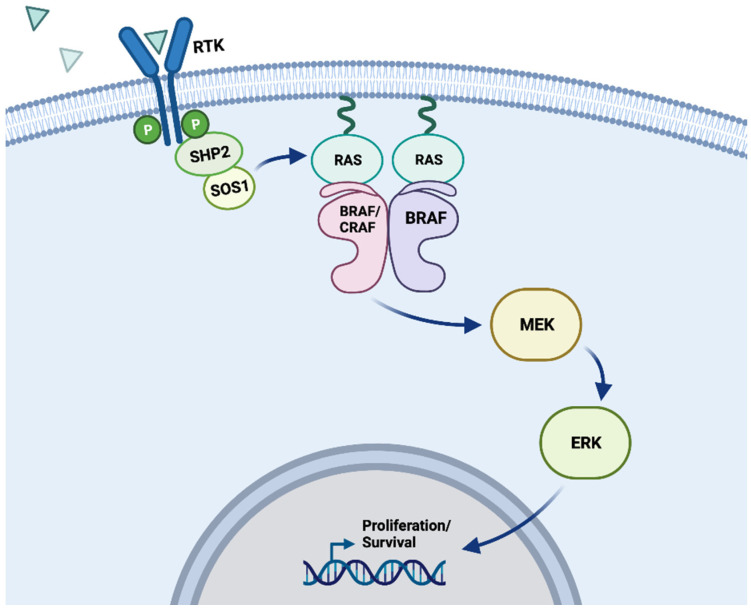
MAPK signaling pathway. Schematic depicting the physiological activation of BRAF within the MAPK signaling pathway. Position of a wild-type BRAF kinase dimer (either BRAF/BRAF or BRAF/CRAF) within the MAPK signaling pathway. Receptor tyrosine kinases (RTKs) receive signaling from ligands (indicated by blue triangles), which then begin a signaling cascade that passes through RAS, RAF, MEK, ERK, and into the nucleus to impact cell proliferation and survival. In a wild-type cell, these processes can be activated and inactivated through regulation pathways that conserve typical metabolic order, but oncogenic mutations to these kinases can disrupt the cell’s ability to control these signals [10]. “Created with BioRender.com”.

Located on chromosome 7q34, V-Raf Murine Sarcoma Viral Oncogene Homolog B (*BRAF*) codes for a serine/threonine protein kinase (BRAF), which belongs to the rapidly accelerated fibrosarcoma (RAF) protein family that also includes ARAF and CRAF [1]. These proteins are direct activators of the mitogen-activated protein (MAP) kinase/extracellular signal-regulated kinase (MEK)/extracellular signal-regulated kinase (ERK) signaling pathway as well as effectors of rat sarcoma (RAS) proteins (Figure 1) [2,3,4,5,6,7,8,9]. Importantly, this pathway plays a key role in cell growth and fate in normal cells as well as in cancerous cells.

**Figure 2:**
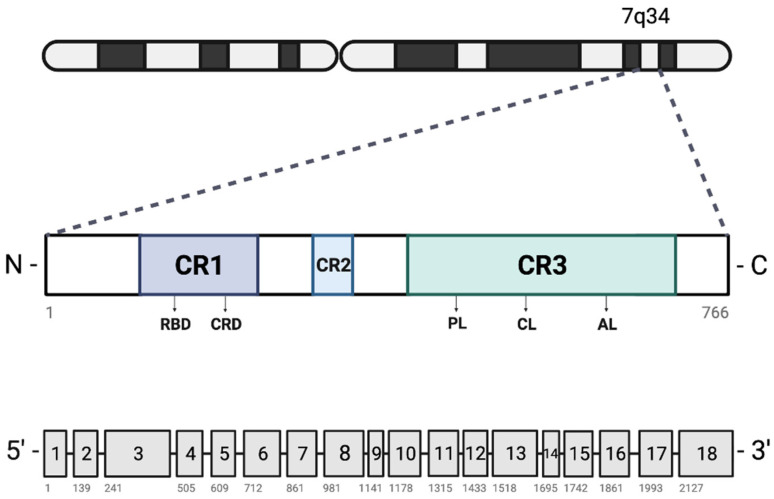
Secondary structure of BRAF. [**Top**] Locus of BRAF gene on chromosome 7q34. [**Middle**] Secondary structure of the BRAF protein from amino acid 1 to 766, with conserved regions (CR) 1–3 and functional domains including the RAS-binding domain (RBD), cysteine-rich domain (CRD), phosphate binding loop (PL), catalytic loop (CL), and activation loop (AL). [**Bottom**] Representation of the 18 exons that make up BRAF, with the number of each exon’s first base pair specified next to the gene (only counting the translated sequence, omitting UTR and flanking sequences). “Created with BioRender.com”.

RAF proteins contain three conserved regions (CR) with differing functionalities (Figure 2). The CR1 domain is divided into two subdomains: a cysteine-rich domain (CRD) involved with RAF kinase domain autoinhibition and needed for RAS protein interaction, and a RAS-binding domain which serves as the interface for RAS proteins [11]. The CR2 domain functions as an inhibitor of RAS protein binding and RAF activation [12] and the CR3 domain possesses serine and threonine-mediated kinase activity [13].

**Figure 3:**
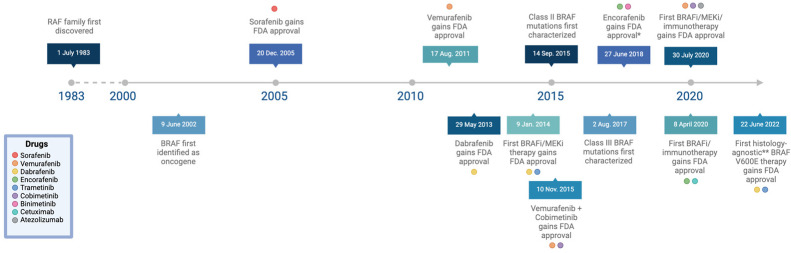
Timeline of key advancements in the understanding of BRAF mutations: breakthroughs in research, FDA approvals for novel treatment options, and the discovery of the three classes of BRAF mutations [15,16,17,18,19,20,21,22,23,24,25]. (* Encorafenib not approved for monotherapeutic treatment, only in combination with other drugs.) (** Histology-agnostic except for BRAF V600E-mutant CRC.) Adapted from “Icon Pack—Timelines (Horizontal)”, by BioRender.com (2024). Retrieved from https://app.biorender.com/biorender-templates.

Early experiments investigating the role of BRAF in embryonic development showed that targeted disruption in the BRAF gene leads to an embryonic lethal phenotype due to vascular defects during mid-gestation [14]. Unlike ARAF or CRAF knockout mice, BRAF knockout mice display significantly enlarged blood vessels, an increased number of endothelial precursor cells, and apoptotic death of endothelial cells [14]. This revolutionary study was the first to establish BRAF as a vital signaling factor for the development of the vascular system and, in 2002, the first evidence of an association between *BRAF* gene mutations and human cancer was described [15] (Figure 3).

**Figure 4:**
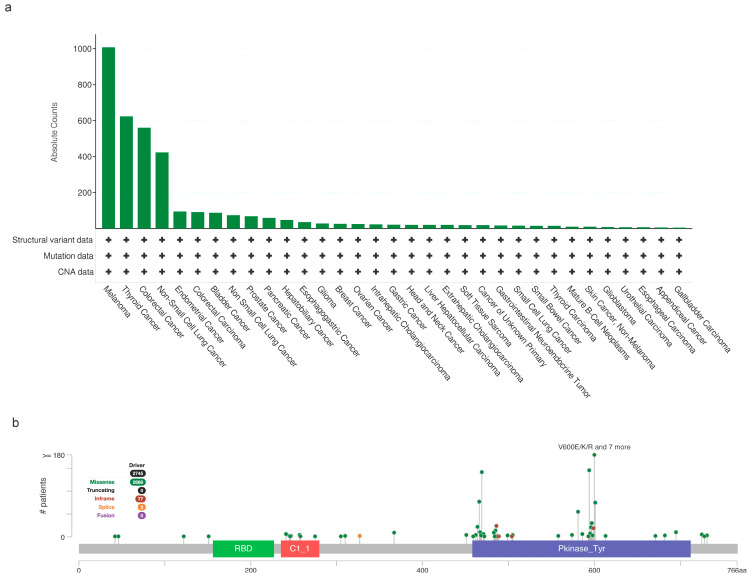
Frequency and types of BRAF alterations across solid tumors. (**a**) Prevalence of BRAF mutations across various cancer histologies (presented as absolute counts). Only published studies on solid tumors with >100 samples, available on cBioPortal [33], were included. The frequency of BRAF mutations varies from around 1% in cancer types such as esophageal, appendiceal, and gallbladder cancers to up to 35% in melanoma. (**b**) Lollipop diagram depicting BRAF mutations identified in several cancer types. The most prevalent mutation, V600E, is localized in the kinase domain and has been frequently reported in melanoma, thyroid, colorectal, and non-small-cell lung cancer. BRAF mutations that mapped in the RAF-like RAS binding and C1 domains are also depicted. These mutations are very rare, their oncogenic potential is mainly unclear, and they are found in a variety of histologies.

Since then, *BRAF* mutations currently account for approximately 7% of all human solid tumors. Notably, BRAF mutations are seen at particularly high rates in melanoma, colorectal cancer (CRC), lung cancer, and papillary thyroid carcinomas (PTCs) [15,26,27,28,29,30,31] (Figure 4a). Specifically, BRAF-activating mutations are primarily limited to the kinase domain, encompassing exons 11 to 15 [32] (Figure 4b). Among the various *BRAF* mutations, exon 15 p.V600E is by far the most prevalent [15] (Figure 4b).

Given over 200 *BRAF*-mutant alleles have been identified in human tumors [25], these mutations have been divided into three classes depending on the activity of the BRAF protein present [25,31,34,35,36].

**Figure 5:**
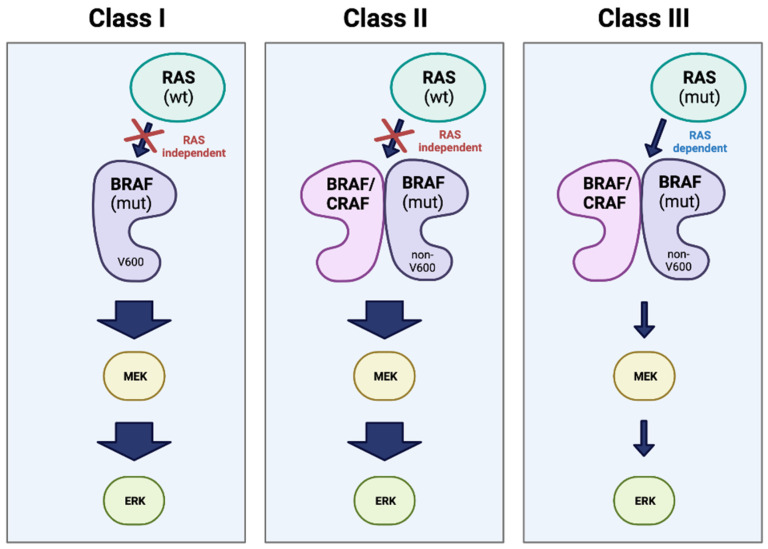
Classes of BRAF mutation. Representation of the three major classes of oncogenic mutations in BRAF. Class I involves a RAS-independent, kinase-active BRAF monomer with a V600 mutation. Classes II and III are non-V600 mutations, and both act as dimers with either CRAF or a second BRAF. However, Class II is RAS-independent and results in high kinase activity, while Class III is dependent on mutant RAS and results in little to no kinase activity (represented in the figure by arrow thickness) [25,37,38]. “Created with BioRender.com”.

Of the three classes of mutations, class I is the most common given it includes exon 15 p.V600 alterations. By inducing elevated levels of kinase activity, these mutations lead to the activation of the MEK/ERK pathways in a manner that is independent of protein dimerization and RAS activation [25,31,34,35,36]. Class II mutations, like class I mutations, are RAS-independent and include gene fusions as well as various point mutations (exon 11 p.G464E/V, exon 11 p. G469A/R/V, exon 15 p. L597Q/V, and exon 15 p.K601E/N/T). However, unlike class I mutations, class II alterations require protein dimerization to induce MEK/ERK pathway activation and they promote both intermediate and high kinase activity [25,31,34,35,36]. In contrast, class III mutations are RAS-dependent and exhibit both increased affinity for RAS-GTP as well as increased binding with CRAF when compared to BRAF wild-type (WT), leading to hyperactivation of the MEK/ERK pathway [25] (Figure 5).

## 2. BRAF Mutations in Cancer

### 2.1. Melanoma

*BRAF* mutations are highly prevalent in melanomas, ranging from approximately 40% to 60% of all reported cases [39]. Of these alterations, 97% occur at exon 15 (codon 600) [40] with up to 90% being just the p.V600E mutant. This mutant arises from a transverse mutation in nucleotide 1799 turning a T to an A (c.1799T>A), ultimately resulting in the valine to glutamic acid substitution (p.V600E) observed [41]. From an epidemiology standpoint, *BRAF*-mutant melanomas often occur in younger patients, frequently possessing a superficial diffusion or nodular morphology. Unlike WT cases, *BRAF*-mutant melanomas are more prone to brain metastasis [26,42] and are often localized in areas with limited sun damage [43].

Initially, tumors harboring the V600E mutation were not treated differently than those with *BRAF* WT. However, given the significantly shorter response to conventional chemotherapy/radiotherapy, a shift in treatment approach was necessary.

Thus came the rise of RAF inhibitors, developed to explore the possibility of targeted therapy in this context. With the onset of targeted therapies, clinical trials such as coBRIM or COLUMBUS revealed the efficacy of specialized BRAF inhibitors in cases of *BRAF* exon 15 p.V600-mutant melanomas [44,45]. In addition to V600E, other mutations observed at this codon are p.V600K, accounting for about 10% of cases, p.V600R (1%), p.V600M (0.3%), or p.V600D (0.1%) (Figure 4b) [41].

Though there is abundant evidence supporting the efficacy of BRAF inhibitors against the most common mutants, their effect in rarer cases has yet to be established [46]. In addition, resistance to therapy has begun to emerge in clinics. To overcome these limitations, combinatorial therapies including RAF and MEK inhibitors as well as immunotherapy have recently been explored with promising preliminary results. The COMBI-d (NCT01584648) clinical trial was the first BRAF-mutant melanoma-focused trial to evaluate the efficacy of the combination of dabrafenib plus trametinib against dabrafenib alone. Upon a 36-month follow-up, the PFS of the combination group was 22% against 12% for the dabrafenib monotherapy arm [47]. Additionally, in the phase 3 clinical trial CheckMate 067, a 6.5-year follow-up analysis revealed that the combination treatment of nivolumab and ipilimumab worked better than nivolumab alone in patients with BRAF-mutant melanoma (6-year PFS 38% vs. 23% and 6.5-year overall survival (OS) of 57% vs. 43%) [48]. Currently, the standard of care for the treatment of BRAF V600E-mutant melanoma includes a combination of vemurafenib (RAFi) plus cobimetinib (MEKi) plus atezolizumab (anti-PD-L1 mAb; Table 1) [49].

### 2.2. Thyroid

In general, *BRAF* gene mutations occur more frequently in sporadic papillary thyroid carcinomas (PTCs) such as aggressive microcarcinomas and tall-cell variant cancers in adult patients [50,51], followed by poorly differentiated carcinomas and PTC-derived anaplastic thyroid carcinomas (ATCs) [52,53,54,55]. In other thyroid lesions such as follicular or medullary carcinomas as well as benign neoplasms, *BRAF* mutations are very rare or never seen [52,53,54,55]. However, in general, the presence of BRAF V600E has been associated from around 18 to 87% of thyroid cancers [54,56].

Through both in vitro and in vivo work, it was believed that the V600E mutation in particular associates with an invasive thyroid cancer phenotype and promotes thyroid cancer progression [57,58]. However, a consensus on whether this can be confirmed has not been reached. Nevertheless, the presence of the *BRAF* exon 15p.V600E point mutation does play a critical role in the diagnostic and prognostic outlook in patients [59,60]. In addition to associated poorer outcomes and aggressive behavior [51,61,62,63] when compared to WT, *BRAF* mutations have been linked to a higher risk of disease recurrence and persistence [63,64]. Furthermore, other non-BRAF factors such as extrathyroidal tumor invasion, older age, lymph node and distant metastases, and being male have all been tied to poorer prognostic outcomes [53,63,65,66,67,68,69,70].

### 2.3. Lung Cancer

From the first report of a *BRAF* mutation in non-small-cell lung cancer (NSCLC) in 2011 [28], variable mutation frequencies have been reported ranging from 1.5–3.5% to 7–8% [34,71,72,73]. Among the mutations identified, class I alterations are the most prevalent, though other mutations have been reported [74]. Importantly, nearly all cases of *BRAF*-mutant NSCLC display strong expression of thyroid transcription factor 1 (TTF-1) and feature an adenocarcinoma morphology with a papillary growth pattern [28]. While *BRAF* mutations are primarily associated with a glandular morphology, some cases have been reported of these alterations in small cell carcinoma as well as varying NSCLC subtypes ranging from pulmonary sarcomatoid carcinoma to squamous cell carcinoma [75,76,77].

Given the variability in the hypothesized epidemiological factors affecting the frequency and distribution of *BRAF* mutations in NSCLC, molecular testing through next-generation sequencing (NGS) provides the best prognostic tool for their identification [78]. In some cases, such as with smokers harboring *BRAF*-mutant lung cancer, the presence of such a mutation is associated with longer median progression-free survival (PFS) when compared to non-mutant groups when treated with immune checkpoint inhibitors. In addition to prognostic value, *BRAF* mutations serve as positive predictive markers for the identification of NSCLC patients who could potentially benefit from targeted therapy [79,80,81,82]. For instance, in the lung cancer cohort of the phase II, basket, open-label AcSé trial, an objective response was observed in 43/96 patients in the BRAF V600-mutant group, while no objective response was reached in the BRAF non-V600 arm. Thus, in the case of BRAF V600-mutant NSCLC, combination therapies involving BRAF and MEK inhibitors such as dabrafenib + trametinib or encorafenib + binimetinib (currently standard of care for the management of these tumors) are effective clinical options (Table 1) [83,84].

### 2.4. Colorectal

Slightly more prevalent than in lung cancer, *BRAF* mutations are found in about 10% of CRCs [85,86]. From an epidemiology standpoint, *BRAF* mutations in CRCs are more commonly detected in patients aged 70 or older and in women. As for their morphology, *BRAF*-mutant CRCs are typically localized in the proximal colon, displaying a poorly differentiated histology characterized by serrated and mucinous components [87,88]. In contrast to the other cancer types discussed, in CRCs, non-p.V600E mutations occur at higher frequencies than *BRAF* p.V600E in younger patients and men, are located on the distal colon, display low-grade histology, and have a longer median OS in response to chemotherapy [89]. Lastly, *BRAF*-mutant CRCs often display high microsatellite instability (MSI-H) as well as a high CpG island methylator phenotype (CIMP-H) [90,91,92]. Specifically, 30–50% of patients with sporadic MSI-H also possess a BRAF mutation [93]. In prognostic terms, CRC patients possessing BRAF exon 15 p.V600E mutations display lower DFS, OS, and cancer-specific survival (CSS) when compared to non-BRAF-mutant CRC regardless of disease stage or chemotherapeutic intervention [94]. Unlike the other cancer types discussed above, targeted therapy has also shown disappointing results in *BRAF*-mutant CRC with monotherapies being mainly inefficacious and combination therapies, including immunotherapy-based regimens, achieving ORR in just about 30% of patients [95]. Currently, the approved therapy for BRAF V600E CRC includes a combination of the second-generation RAF inhibitor encorafenib plus the anti-EGFR antibody cetuximab (Table 1) [49].

### 2.5. Other Cancers

Histiocytic neoplasms such as Langerhans cell histiocytosis (LCH) and Erdheim–Chester disease (ECD) are derived from macrophage/dendritic lineages and known to be enriched for BRAF V600 mutations with frequencies up to 50% [96,97]. As such, clinical trials like the VE-BASKET study (NCT01524978), which evaluated the efficacy of vemurafenib in nonmelanoma cancers harboring BRAF V600 mutations, revealed that these mutants are highly targetable with an ORR of 61.5% in the larger cohort and an ORR of 54.5% in the ECD cohort [96].

Oncogenic mutations in BRAF are also seen at low frequencies in other cancer types (Figure 4a). For example, pediatric low-grade gliomas (PLGGs) often harbor the *BRAF* p.V600E point mutation. Importantly, patients with mutated tumors have poorer clinical outcomes than patients presenting with BRAF WT disease [98,99]. For example, in a longitudinal study conducted in Ontario in which PLGG patients were treated with a combination of chemotherapy and radiation therapy, 69 of 405 of them were found to harbor the BRAF V600E mutation. This cohort exhibited a 10-year PFS of 27% compared to 60.2% in the BRAF WT group [99]. In contrast, the presence of the *BRAF* exon 15 p.V600E has no impact on prognosis in patients with glioblastoma, even when tumors harbor epithelioid morphology without isocitrate dehydrogenase (*IDH*) alterations [100].

Though frequencies are low, *BRAF* mutations have also been reported in both breast cancer and ovarian cancer [101].

## 3. Diagnostic Approaches for BRAF Mutations

### 3.1. Detection of Mutations

With the advancements in diagnostic technologies, a wide range of methods exist for the detection and diagnosis of *BRAF*-mutant cancers. Among these are sequencing-based and PCR-based methods as well as immunohistochemistry (IHC) [102,103]. Additionally, the onset of liquid biopsy testing technologies has allowed for minimally invasive genetic testing, giving the opportunity to evaluate clonal evolution as well as resistance mechanisms through the course of treatment [37,104,105,106,107]. The shift from genotyping focused on few genes with sequential testing through single-gene assays to next-generation sequencing (NGS), which scans a larger set of genes, has allowed for the detection of previously undiscovered alterations as well as increasing the likelihood of detecting rare alterations [74]. As for more frequently studied genes, NGS provides the opportunity to distinguish uncommon genotypes, which are often overlooked in hotspot PCR-based assays [108]. Furthermore, NGS allows for the identification of several classes of alterations such as fusions, amplifications, and mutations [109,110], as well as offering the potential to unveil novel alterations which have not yet been discovered.

Though DNA-based NGS is usually the main means for genotyping, its variable sensitivity to alternatively spliced transcripts and fusions renders it rather unreliable for the analysis of minute changes [111,112]. However, the exploration of RNA-based methods has allowed for the direct assessment of oncogenic RNA transcripts lacking large intronic sequences. This has enabled more sensitive and efficient analyses, leading to the detection of occult kinase fusions which are often overlooked by DNA sequencing [111,112]. In addition to being more efficient, RNA-based analysis allows for higher specificity through confirmation that some fusions produce novel chimeric transcripts while others of uncertain significance detected in DNA are not transcribed into oncogenes [113]. Given RNA sequencing can directly capture aberrant splicing byproducts, it largely outperforms DNA hybrid capture-based target enrichment, allowing for the determination of which variants lead to certain exons being skipped [114]. Though RNA-based sequencing offers many advantages when compared to DNA-based assays, RNA is often prone to clinical testing failure given its predisposition to degradation and lability [115,116]. Regarding oncogenic hotspot mutations, a DNA-based technique should be sufficient for a reliable diagnosis. RNA-based assays are instead preferable for detecting BRAF fusions which occur at low frequencies in melanoma (3%), glioma (2%) thyroid cancer (1%), pancreatic carcinoma (0.3%), NSCLC (0.2%), and colorectal cancer (0.2%) [117]. Another alternative is IHC utilizing the anti-BRAF V600E (VE1) mouse monoclonal antibody, which was generated to specifically recognize the mutated amino acid sequence from amino acids 596 to 606 [118]. Given IHC equipment is both widely available in pathology laboratories and gives much faster results than molecular biology techniques, the VE1 IHC diagnostic method provides a great alternative to conventional BRAF V600E genotyping, particularly for those tumor types in which this mutation is frequently found (e.g., melanoma, histiocytic neoplasms).

While adequate tumor tissue samples are pivotal to successful NGS, they are often not enough for comprehensive testing and collecting them can be invasive for the patient [119]. Through the use of around 3–10 mL of plasma followed by the analysis of circulating tumor DNA (ctDNA), liquid biopsies can provide additional information supplementing tissue-based approaches or replace them altogether in certain instances [120]. While they serve as an alternative approach, liquid biopsies require ultra-deep sequencing (>10,000× depth) and often include fewer genes than tissue-based panels in order to balance sequencing depth and breadth [121]. This, along with the fact that ctDNA in plasma is rather scarce, results in the exclusion of less commonly altered genes as those alterations which are highly recurrent are prioritized.

### 3.2. Associated Biomarkers

In the case of CRC, MSI-H status may be indicative of the presence of a BRAF mutation, with co-occurrence of the two often leading to later presentation of disease as well as poorer prognosis [122]. BRAF mutations themselves have been identified as important biomarkers and are often used to predict a therapeutic response. In some cases such as advanced melanoma, the presence of a BRAF mutation actually improves patient prognostic outlook [123], while in other types such as colorectal cancer, the finding of a BRAF V600E mutation is associated with resistance to standard therapy and is overall a biomarker of poor prognosis [124].

The prevalence of BRAF mutations in certain cancer types varies by a range of factors including age. In some cancers such as advanced melanoma, V600 mutations occur at particularly higher rates in adolescents and young adults when compared to older adults (68% vs. 46%, *p* < 0.001) [125], identifying age as a potential biomarker for the presence of the BRAF V600E mutation in these tumors. As such, the therapeutic approach may vary between the two populations, with younger patients with advanced melanomas more likely than older adults to receive targeted therapy upfront based on the presence of the V600 mutation. Given this, it appears that in the case of BRAF-mutant cancers, treatments are influenced by the presence and type of BRAF mutation rather than directly by age, though precise regimens may differ by population type [126].

## 4. Targeted Therapies for BRAF-Mutant Cancers

Before delving into the currently available FDA-approved therapies for the treatment of BRAF-mutant tumors (summarized in Table 1), it is important to understand how BRAF inhibitors interact with the BRAF kinase itself. Like most kinases, RAFs are composed of two domains: the N-terminal and C-terminal lobes [127]. These domains are held together by a flexible hinge, and the cleft between them acts as an active site that can bind to substrates such as ATP and kinase inhibitors. Two regulatory elements of BRAF, the αC-helix and the DFG motif, are particularly relevant to the function of its inhibitors [128]. Under typical conditions, these elements can switch between inactive-OUT and active-IN conformations, but treatment with RAF inhibitors can cause allosteric structural changes that lock specific conformations into place [24,129]. These conformations can be used to categorize BRAF inhibitors into type I (αC-helix-IN/DFG-IN), type I_1/2_ (αC-helix-OUT/DFG-IN), and type II (αC-helix-IN/DFG-OUT) [129]. BRAF inhibitors can also be categorized by generation, with first-generation inhibitors being the least specific treatment and third-generation ones being the most recent developments.

### 4.1. First-Generation BRAF Inhibitors

First-generation RAF inhibitors were created prior to the discovery of the oncogenicity of BRAF mutations and were initially designed to act as ATP-competitive inhibitors of CRAF [130]. These first-generation drugs stabilize the αC-helix into the active-IN position [131]. Although several iterations of this class of drug have gone through preclinical trials, sorafenib is the only first-generation RAF inhibitor to have gained FDA approval [132]. This particular inhibitor also causes an inactive-OUT conformation of the DFG motif, meaning that it is classified as a type II inhibitor [133]. Sorafenib is not particularly effective against cells that exhibit BRAF V600E mutations; even so, its multikinase inhibition activity makes it effective enough to have been FDA-approved to treat advanced renal cell carcinoma (median overall survival [mOS] 17.8 months for the sorafenib branch vs. 15.1 months for the placebo, median progression-free survival [mPFS] 5.5 vs. 2.8 months), unresectable hepatocellular carcinoma (mOS 10.7 vs. 7.9 months, mPFS 5.5 vs. 2.8 months), and metastatic differentiated thyroid carcinoma (mOS 42.8 vs. 39.4 months, mPFS 10.8 vs. 5.8 months) [16,134,135]. Even with its nonspecific approach to treatment, this pioneer drug helped break ground on a whole new approach to the treatment of BRAF-mutant cancers.

### 4.2. Second-Generation BRAF Inhibitors

Upon the discovery of BRAF mutations and their roles as oncogenes in 2002 by Davies et al., researchers began to develop more targeted therapies in the form of second-generation RAF inhibitors [134]. These drugs focus on inhibiting BRAF V600E, a mutation that is particularly prevalent in melanoma, thyroid cancers, and colorectal cancers [15,134]. Specifically, they bind the inactive-OUT position of the αC helix [54]. Second-generation RAF inhibitors can also be effective against other Class I BRAF mutations, which include all V600 variants and make up over 90% of all BRAF mutations [32]. Class I BRAF mutations are highly kinase-active, RAS-independent, and signal as monomers [136]. Vemurafenib became the first of these inhibitors to gain FDA approval in 2011, specifically as a monotherapy for the treatment of unresectable or metastatic melanomas with V600E mutations [25]. Currently, it is also used to treat BRAF V600-mutant cases of Erdheim–Chester disease [17]. Dabrafenib, which was FDA-approved as a treatment for unresectable or metastatic melanomas with V600E mutations in 2013, has a similar mechanism of action as vemurafenib and also inhibits the BRAF monomer [17]. These monotherapeutic approaches have been vital in the treatment of patients with Class I BRAF mutations. However, emerging resistance to these drugs—as well as the need for a broader range of treatment options—has led to the advent of several combination therapies [137].

### 4.3. Combination Therapies

A key issue with BRAF inhibitor monotherapy is that it can sometimes lead to increased activation of MEK and ERK, which are downstream of BRAF in the MAPK pathway [104]. This can reduce the efficacy of treatments and even cause secondary skin cancers to occur in some patients [138]. The development of combination therapies pairing BRAF and MEK inhibitors is meant to bypass this effect and has led to more targeted treatments for a wider range of histologies. For example, dabrafenib in combination with the MEK1/2 inhibitor trametinib was first approved by the FDA in 2014 for the treatment of unresectable or metastatic melanomas with BRAF V600E and V600K mutations [139]. Currently, this combination is also approved to treat a variety of BRAF V600E-mutant cancers of different histologies including non-small-cell lung cancer (NSCLC) with the involvement of lymph node(s), locally advanced or metastatic anaplastic thyroid cancer, pediatric low-grade glioma, and any unresectable or metastatic solid tumors with no other satisfactory treatment options. The latter of these approvals, which was granted in 2022, is an especially relevant breakthrough as it represents the first histology-agnostic treatment option for patients with BRAF V600E-mutant solid tumors [49].

Similarly, the combination of vemurafenib and the MEK1/2 inhibitor cobimetinib has been approved for the treatment of unresectable or metastatic melanomas with BRAF V600E and V600K mutations [18]. Despite not having been approved for monotherapeutic use, the second-generation BRAF inhibitor encorafenib is FDA-approved for the treatment of unresectable or metastatic melanomas with BRAF V600E and V600K mutations [140]. The combination of encorafenib and the anti-EGFR antibody cetuximab, for instance, was FDA-approved in 2020 to treat metastatic colorectal cancer with a BRAF V600E mutation after prior therapy. Combination therapies can also involve three drugs, such as in the case of vemurafenib, cobimetinib, and the PD-L1 blocking antibody atezolizumab; this trio was FDA-approved for treating unresectable or metastatic melanomas with any BRAF V600 mutation [19]. Dual BRAF and MEK inhibition is already known to improve immunity responses against tumors, so the addition of these immunotherapeutic drugs is meant to provide an additional synergistic effect for certain histologies [141]. The use of antibodies in the treatment of BRAF-mutant cancers is reflective of a growing interest in immunotherapy, both within the field of oncology and beyond it.

### 4.4. Third-Generation BRAF Inhibitors

Despite having promising results in the treatment of Class I BRAF mutations, second-generation RAF inhibitors are not nearly as effective against Class II and III mutations [142]. This is due to a key structural difference in the expression of these mutations; while Class I BRAF functions as a monomer, Class II and III act as homo- or hetero- and heterodimers, respectively [143]. Second-generation BRAF inhibitors such as vemurafenib and dabrafenib bind to the BRAF kinase, stabilizing the αC-helix into its inactive OUT position. This is an effective approach to inhibiting the BRAF monomers associated with Class I BRAF mutations. However, in dimers, this same αC-OUT position causes a steric strain between the inhibitor and kinase that leads to a negative allosteric effect in the second protomer [144]. As a result, the monomer-targeting second-generation inhibitors fall flat in the inhibition of dimerized non-V600 mutations. There are currently no FDA-approved BRAF inhibitors that adequately inhibit Class II and III mutations, but several third-generation BRAF inhibitors are being developed and tested in clinical trials in order to meet this need. Third-generation BRAF inhibitors are characterized by their targeting of both monomers and dimers of the BRAF kinase [142]. One example of this broader approach to treatment is the use of αC-IN RAF inhibitors, such as CEP-32496 and RAF-265 [89,145]. These drugs help to reduce steric hindrance, inhibiting both RAF protomers and reducing the overall activity of dimeric RAF [146]. Unfortunately, this same αC-IN feature also induces RAF priming, which leads to a significant increase in RAF dimerization [144]. αC-OUT inhibitors do not catalyze RAF priming to the same degree, but in cases of heightened activation of the upstream RAS, they can cause RAF priming and dimerization to increase through an effect known as inhibitor-induced paradoxical activation [147]. Future third-generation BRAF inhibitors will have to target both monomeric and dimeric BRAF, as well as avoiding paradoxical activation both upstream and downstream of BRAF.

### 4.5. Current Efforts in Targeting Resistance to Therapy

In response to the prevalence of patients with BRAF mutations developing resistance to treatments through the paradoxical re-activation of MEK/ERK detailed above, researchers have been working on a new class of RAF inhibitors referred to as “paradox breakers” [49]. These drugs, which were designed by making structural modifications to vemurafenib, are meant to lessen the re-activation effect that would otherwise allow tumors to develop resistance to treatment [49]. Two promising examples of this are plixorafenib and PLX904; they not only inhibit monomeric BRAF V600E-mutant cancers but can also inhibit BRAF homodimers and BRAF-CRAF heterodimers [144,148]. Plixorafenib is already undergoing testing in a phase I/IIa clinical trial on patients with BRAF class I and II mutated solid tumors [NCT02428712]. Unfortunately, these two drugs have not been found to be effective inhibitors of CRAF homodimers and ARAF dimers, which means they will most likely not be effective against class III BRAF mutations [49,149,150]. Even so, they are capable of inhibiting a wide range of targets and offer physicians a novel approach to treatment [49,149,150].

Another important aspect of treating resistance to therapy is having effective tools to use if a patient’s tumor spreads beyond the organ in which it originated. Brain metastases are particularly common for patients with melanoma, which is one of the major histologies in which BRAF mutations are found [151]. A new brain-penetrant RAF inhibitor called Compound 1a is currently being tested in preclinical research as a potential treatment option for these metastases, and is already showing promising results compared to approved BRAF and MEK inhibitors [152,153].

## 5. Resistance Mechanisms and Overcoming Challenges

### 5.1. BRAF and the MAPK Signaling Pathway

BRAF, whether wild-type or mutated, is an inextricable part of the larger MAPK signaling pathway. This means that any attempt at targeting it without disrupting other parts of the pathway can present a challenge. For example, one of the most common resistance mechanisms that tumors develop against BRAF inhibitors is the reactivation of various components of the MAPK pathway [154]. Monotherapeutic administration of BRAF inhibitors often leads to the reactivation of the MEK/ERK effector cascade, which is downstream of BRAF [155]. This is why BRAF/MEK inhibitor combination therapy has become prominent in research efforts and has led to significant improvements in patient response rates, progression-free survival, and median duration of response when compared to monotherapy [156]. As with monotherapy, the administration of combination therapies is also subject to the emergence of various mechanisms of resistance. Most of these mechanisms are again MEK/ERK-dependent, although MEK/ERK-independent variations have also been identified [157].

### 5.2. MEK/ERK-Dependent Resistance

MEK/ERK-dependent forms of BRAF inhibitor resistance can be further subdivided based on whether they are adaptive or acquired. Adaptive resistance occurs without new mutations arising, and its prevalence varies by tumor histology due to the unique variations in receptor expression that these cancers already possess [158]. For example, EGFR is active in both colon cancer and melanoma, but the comparatively higher levels of EGFR expression in colon cancer cause it to develop drug resistance significantly faster than in melanoma. In a similar mechanism involving HER2 and HER3 ligands, V600E thyroid cancers are able to form resistance to vemurafenib more quickly than melanoma can [159,160]. These variations in resistance highlight the importance of taking tumor histology into account when considering treatment options for BRAF-mutant cancers.

In contrast to adaptive resistance, acquired MEK/ERK-dependent resistance arises when changes are made to actual molecular switches of the MAPK pathway [161]. These changes occur as a result of selective pressures caused by treatment with kinase inhibitors. Dependent resistance can arise through alterations directly at the BRAF level, or it can begin upstream or downstream of BRAF such as at RAS, MEK1/2, and ERK 1/2 [162,163]. Similar to adaptive resistance, acquired MEK/ERK-dependent resistance can vary based on tumor histology [164]; this mechanism is more commonly found in skin, colon, and thyroid cancers, though it has also been found to occur in pancreatic, lung, and ovarian tumors [161].

As part of acquired resistance to BRAF inhibitors, emerging alterations in the BRAF gene itself can also occur [165]. For example, the amplification of BRAF has been observed in dabrafenib and trametinib combination therapy-resistant melanoma and colon cancer patients, with additional variations in BRAF splicing found in the melanoma patients [163,165,166,167]. Of the dabrafenib and trametinib-resistant melanoma patients, one out of five were found to have a BRAF splicing alteration associated with a lack of exons 2–10 [163]. A rarer alteration, found in 0.4% of this same subset of dabrafenib and trametinib combination therapy-resistant melanomas, possessed in-frame deletion mutations related to exons 2–8 that are associated with the RAS-binding domain [168]. Deleterious mutations resulting in BRAF activation were also found in 0.6–1% of lung, pancreatic, thyroid, and ovarian cancers [161,169]. These mutations lock the αC-helix into the active-in conformation by shortening the β3/αC-helix loop, thereby facilitating the formation of BRAF dimers [161,169]. Lastly, a β3-αC deletion mutation has been found to diminish the binding abilities of BRAF inhibitors AZ628, dabrafenib, and vemurafenib by increasing the flexibility of the αC-helix [170]. These relatively novel findings show that not all acquired resistance to BRAF inhibitors appears upstream or downstream of BRAF, and that some alterations are capable of directly affecting the structure and function of BRAF itself.

### 5.3. MEK/ERK-Independent Resistance

Although it is comparatively less common, MEK/ERK-independent resistance can also arise due to treatment with BRAF inhibitors [157]. Preclinical research has helped expand our current understanding of this category of resistance, with mechanisms ranging from the loss of phosphatase and tensin homolog (PTEN) to changes in metabolic processes [164,171]. However, not much is known about MEK/ERK-independent resistance mechanisms in the context of BRAF/MEK inhibitor combination therapy, making this an important area for future research to target [159].

### 5.4. Potential New Targets for Overcoming Mechanisms of Resistance

Along with investigating BRAF and MEK/ERK in the treatment of BRAF-mutant cancers, researchers are looking into the role of other factors that may affect the emergence of resistance to BRAF inhibitors. For example, the stem cell-associated transcriptional factor POU4F1 was found to play a part in the development of malignant BRAF-activated tumors [172]. In melanoma, POU4F1 is also able to promote acquired resistance to vemurafenib through paradoxical re-activation of MEK/ERK [172]. These findings suggest that POU4F1 could be a potential new target in the treatment of BRAF-mutant melanoma, but further studies must be conducted in order to determine its therapeutic potential.

Another potential target, a PTEN pseudogene transcript called PTENP1-AS, is expressed in high levels in BRAF inhibitor-resistant melanoma [173]. When PTENP1-AS was targeted by investigators, the melanoma cells were resensitized to the BRAF inhibitors they had previously developed resistance to [173]. This promising relationship shows potential for future use, both as a diagnostic measure and as a method of overcoming drug resistance.

There has also been some headway on incorporating theranostic treatment options, such as photodynamic therapy (PDT), into the arsenal of approaches that can be used to treat BRAF-mutant cancers [174]. PDT involves activating a photosensitizer with a specific wavelength of light, which then interacts with molecular oxygen to produce reactive oxygen species that kill targeted cells [175]. The specificity of PDT could prove to be useful in the treatment of BRAF-mutant cancers, especially in combination with other treatment strategies. Antibody–drug conjugates can also be used in the context of BRAFi resistance tumors to target specific antigens found on tumors, which help to reduce adverse effects in apoptosis-resistant histologies such as melanoma [174].

## 6. Clinical Implications and Patient Outcomes

Understanding the various pathways and molecular mechanisms that affect BRAF is crucial for understanding diseases associated with its mutations. However, it is the clinical trials in which these concepts are put into practice that actually dictate their translational value in patient care. Clinical trials on BRAF-mutant cancers and associated conditions have been the basis on which FDA approvals, new standards of treatment, and novel approaches to research have been made. A table summarizing clinical trials to date concerning BRAF mutations, further subdivided by phase classification, is presented in Supplementary Table S1.

Another crucial part of patient care is understanding how well treatments can be tolerated, as this factor often dictates whether or not a person can continue medication. Often, adverse effects are dictated by how many off-target effects a drug causes, so more targeted treatments are typically associated with lower toxicity profiles than their more general counterparts [176,177]. BRAF and MEK inhibitors target their respective kinases, but their close association with the larger MAPK signaling pathway means that these treatments can impact several outcomes of cellular signaling, including cell proliferation, differentiation, and even apoptosis [178,179,180]. These effects can manifest as a variety of different symptoms in varying intensities depending on histology, dosage, mutations, and the specific drug being used [181]. For example, as mentioned previously in this review, the monotherapeutic use of BRAF inhibitors can sometimes lead to re-activation of the MAPK pathway that can lead to paradoxical oncogenesis [182]. This is the reasoning behind the development of combination therapies, but these too come with their own set of symptoms [57]. Clinical trials often report the tolerability of treatments by grading the adverse effects. However, due to variations in several aspects of clinical trials such as patient populations and types of reporting, these metrics are not meant to be directly compared across studies [183]. It is important that healthcare providers be able to consider how treatments will affect their patients, which is why many studies use meta-analyses of clinical trials in order to find a reliable way to compare treatments [184]. Below is a table summarizing the results of one such meta-analysis on the grades of adverse effects associated with BRAF mono- and combination therapies (Table 2).

## 7. Conclusions and Future Directions

BRAF hotspot mutations are oncogenic drivers in multiple cancer types. While current approaches for treating BRAF V600-mutant solid tumors vary based on cancer type, the FDA granted accelerated approval for the use of BRAFi dabrafenib in combination with MEKi trametinib for the treatment of all unresectable or metastatic BRAF V600E-mutant solid tumors except for CRC in June 2022. For BRAF V600E-mutant CRC, the current standard of care involves targeting BRAF and EGFR through the use of encorafenib in combination with cetuximab. While BRAF inhibitors work very well in the context of class I mutations, they often lose efficacy against class I mutations as a result of ERK reactivation through RAF dimer induction. Though this ERK reactivation can be moderated through the use of vertical pathway targeting (i.e., using a BRAFi in combination with a MEKi), the formation of RAF dimers still poses a formidable challenge in clinical intervention.

In addition, since class II and III BRAF mutations also function as dimers, no approved BRAF inhibitors are currently active against these mutant tumors. As such, BRAF drug discovery seems to be moving in the direction of RAF dimer targeting, beginning with the use of “paradox breakers” designed to avoid the paradoxical induction of ERK signaling. Moreover, the recent inclusion of immunotherapy as part of the standard of care for the treatment of BRAF-mutant melanoma will be instrumental in not only understanding the effect of ERK inhibition on the tumor-immune microenvironment, but also in determining the impact of the combination of anti-PD-1/PD-L1 therapy with MAPK inhibition. Though these advancements have shown great promise, more work is needed to assess its clinical translatability based on biomarkers and patient selection criteria.

In summary, it is clear that our understanding of BRAF and ways to target it is progressing rapidly as a result of a joint collaboration between scientists and medical professionals. In order to continue to advance in this field, it is imperative that this system be maintained to better the lives of patients with BRAF-mutant cancers.

## Figures and Tables

**Table 1:** BRAF inhibitors and combination therapies currently FDA-approved to treat BRAF mutant cancers.

**BRAF Inhibitors**			
**Drug**	**Inhibitor Type**	**Approved to Treat**	**Date approved by the FDA**
Sorafenib (Nexlavar) Also: Sorafenib tosylate	1st-gen BRAFi	Advanced renal cell carcinoma Unresectable hepatocellular carcinoma Locally recurrent or metastatic, progressive, differentiated thyroid carcinoma (DTC) refractory to radioactive iodine treatment	20 December 2005
Vemurafenib (Zelboraf) Also: PLX4032	2nd-gen BRAFi	Unresectable or metastatic melanoma with BRAF V600E mutation Erdheim–Chester disease with BRAF V600 mutation	17 August 2011
Dabrafenib (Tafinlar) Also: GSK-2118436	2nd-gen BRAFi	Unresectable or metastatic melanoma with BRAF V600E mutation	29 May 2013
Encorafenib (Braftovi) Also: LGX818	2nd-gen BRAFi	(Not approved for use as a single agent)	27 June 2018
**Combination Therapies**			
**Drug**	**Inhibitor types**	**Approved to treat**	**Date approved by the FDA**
Dabrafenib + Trametinib (Tafinlar + Mekinist) Also: GSK-2118436 + GSK-1120212	Dabrafenib: BRAFi Trametinib: MEK1/2i	Unresectable or metastatic melanoma with BRAF V600E/K NSCLC with BRAF V600E mutation and involvement of the lymph node(s), following complete resection Locally advanced or metastatic anaplastic thyroid cancer with a BRAF V600E mutation Unresectable or metastatic solid tumors with BRAF V600E mutation and no satisfactory alternative treatment options Pediatric patients 1 year of age and older with low-grade glioma with a BRAF V600E mutation	9 January 2014
Vemurafenib + Cobimetinib (Zelboraf + Cotellic) Also: PLX4032 + GDC-0973	Vemurafenib: BRAFi Cobimetinib: MEK1/2i	Unresectable or metastatic melanoma with BRAF V600E/K	10 November 2015
Encorafenib + Binimetinib (Braftovi + Mektovi) Also: LGX818 + MEK162	Encorafenib: BRAFi Binimetinib: MEK1/2i	Unresectable or metastatic melanoma with BRAF V600E/K Metastatic NSCLC with BRAF V600E	27 June 2018
Encorafenib + Cetuximab (Braftovi + Erbitux) Also: LGX818 + Encorafenib	Encorafenib: BRAFi Cetuximab: monoclonal antibody, EGFR antagonist	Metastatic CRC with a BRAF V600E mutation (after prior therapy)	8 April 2020
Vemurafenib + Cobimetinib + Atezolizumab (Zelboraf + Cotellic + Tecentriq) Also: PLX4032 + GDC-0973 + RG7446	Vemurafenib: BRAFi Cobimetinib: MEK1/2i Atezolizumab: PD-L1 blocking antibody	Unresectable or metastatic melanoma with BRAF V600	30 July 2020

**Table 2:** BRAF inhibitor adverse effects. Overall incidence rate of grade 3 to grade 5 (G3–G5), and any grade, adverse effects of BRAFi and BRAFi + MEK1/2i treatments (0–50% in green, 51–75% in orange, 76–100% in red).

Drug(s)	G3–G5	Any Grade
Vemurafenib (Zelboraf)	51%	94%
Dabrafenib (Tafinlar)	50%	85%
Encorafenib (Braftovi)	68%	99%
Dabrafenib + Trametinib (Tafinlar + Mekinist)	43%	95%
Vemurafenib + Cobimetinib (Zelboraf + Cotellic)	72%	98%
Encorafenib + Binimetinib (Braftovi + Mektovi)	68%	98%

## Supplementary Materials

The following supporting information can be downloaded at: https://www.mdpi.com/article/10.3390/cancers16061215/s1, Table S1: Clinical trials on BRAF mutated cancers organized by phase.
